# Formulation Composition and Process Affect Counterion for CSP7 Peptide

**DOI:** 10.3390/pharmaceutics11100498

**Published:** 2019-09-29

**Authors:** Sawittree Sahakijpijarn, Chaeho Moon, John J. Koleng, Robert O. Williams

**Affiliations:** 1 College of Pharmacy, The University of Texas at Austin, 2409 University Ave, Austin, TX 78712, USA; sawittree.willy@utexas.edu (S.S.); chaehomoon@utexas.edu (C.M.); 2 Affiliation Lung Therapeutics, Inc., 2801 Via Fortuna Way, Austin, TX 78746, USA; jkoleng@alphavektor.com

**Keywords:** counterion, counterion volatilization, volatile compounds, lyophilization, bulking agent, peptide aggregation

## Abstract

Counterions commonly remain with peptides in salt form after peptide purification. In animal and human studies, acetate counterions are a safer and more acceptable choice for peptides than others (e.g., trifluoroacetate counterions). Various salt forms of caveolin-1 scaffolding domain (CSP7) affect counterion volatilization. The development of lyophilized formulations containing volatile compounds is a challenge because these compounds sublimate away during the process. This work aims to investigate the effect of excipients and lyophilization parameters on the preservation of volatile compounds after lyophilization. The peak areas obtained from ^1^H and ^19^F NMR spectra were used to calculate the molar ratio of counterions to CSP7. We found that the pH modifier excipient had the greatest impact on the loss of counterions. By optimizing the molar ratio of bulking agent to CSP7, volatile compounds can be preserved after lyophilization. Higher chamber pressure during lyophilization can lower the sublimation rate of volatile compounds. Moreover, the loss of volatile compounds affects the stability of CSP7 due to the pH shift of reconstituted solutions, thereby causing peptide aggregation. The optimization of the formulation and processing helps preserve volatile compounds, thus minimizing the pH change of reconstituted solutions and maintaining the stability of peptide.

## 1. Introduction

Idiopathic pulmonary fibrosis (IPF) is a chronic, progressive, irreversible, fibrosing disease characterized by defective alveolar epithelial cells which stimulate the migration, proliferation, and activation of mesenchymal cells. Cells of mesenchymal origin form foci referred to as usual interstitial pneumonia (UIP) lesions which secrete excessive extracellular matrix (e.g., collagen), resulting in scars that destroy the lung architecture [1,2]. Incidence has been estimated at 5–16 cases per 100,000 people per year, while its prevalence reportedly ranges from 13 to 20 cases per 100,000 people per year [1,3]. The morbidity rate of IPF is high; 50% of patients die within 3–4 years [3]. Only two treatments (pirfenidone and nintedanib) have been approved by the Food and Drug Administration for the treatment of IPF, due to their ability to slow progression of the disease. However, several studies have reported that neither of these treatments exhibited a clear advantage on mortality outcomes when compared to the placebo [4,5,6]. Furthermore, while current standard of care treatments reduce the incidence of acute exacerbations which often lead to death [7], patients may experience quality of life reducing side effects.

A caveolin-1 scaffolding domain (CSP7; FTTFTVT) is a novel candidate peptide for the treatment of IPF. Caveolin-1 is a critical regulator of signaling pathways involved in lung injury [8,9]. CSP7 can reverse the suppression of urokinase-type plasminogen activators (uPAs) and the uPA receptor involved in alveolar epithelial cell (AEC) apoptosis [10,11]. Moreover, CSP7 also inhibits the tumor suppressor protein p53 and plasminogen activator inhibitor-1 in type II alveolar epithelial (ATII) cells after lung injury, thus preventing apoptosis of the lung epithelium. Through these pathways, CSP7 restores damaged lung epithelium and hinders disease progression [8,10,12,13]. Prior studies have reported the efficacy of CSP7 as a prophylactic therapy in BLM-induced lung injury in mice [8,11,12,13,14]. Therefore, CSP7 is a promising agent for the prevention and treatment of IPF.

A major challenge in the development of peptide-based medicines is their poor chemical and physical stability. Although an aqueous biopharmaceutical therapeutic is preferred due to the ease of preparation and handling by the end user, many biopharmaceuticals are unstable in aqueous solution, thus forming aggregates [15]. Peptide denaturation and aggregation lead to a decrease in therapeutic action such as antibody activity and an increase in immunological response [15,16].

To prolong the shelf life of biopharmaceuticals, lyophilization is commonly used to prepare protein and peptide formulations in a solid state. However, even in the solid state, stress on the formulation can induce chemical and physical degradation of proteins. This stress includes the conditions encountered during the manufacturing process, distribution, storage, and administration [17]. Sugars (e.g., lactose, sucrose, trehalose) and sugar alcohols (e.g., mannitol) have been widely used as cryoprotectants or lyoprotectants, but moisture sorption and the recrystallization of the bulking agent during storage can minimize the stabilizing effects of bulking agents on protein and peptide aggregation [18,19].

Salts of active pharmaceutical ingredients have been used to achieve desirable formulation properties such as increasing aqueous solubility [20]. Salts are formed when a compound that is ionized in solution forms a strong ionic interaction with an oppositely charged counterion. CSP7 was initially synthesized and purified using reversed-phase chromatography, and it was obtained as a trifluoroacetate (TFA) salt. Trifluoroacetic acid is a liquid at room temperature and is used as a protein-solubilizing and chaotropic agent during peptide purification using reverse-phase HPLC [21]. TFA binds specifically to basic positively charged amino acid side chains and the free N-terminal amino group, forming TFA salt [21]. Recently, CSP7 as a trifluoroacetate (TFA) salt was developed as a lyophilized powder [22]. The lyophilized CSP7 TFA formulated with mannitol and DPBS was physically and chemically stable after storage at 25 °C for up to one month and at 5 °C for up to 10 months [22]. CSP7 TFA remained stable after reconstitution and nebulization via the Aeroneb Pro^®^ vibrating mesh nebulizer [22].

Although free TFA ions can be removed using freeze drying [23], the TFA counterions that were directly bound to the peptide molecules can affect biological and physicochemical properties [24]. It was reported that TFA counterions of amylin exhibit less cell proliferation in osteoblasts, as compared to hydrochloride counterions [25]. Additionally, studies have reported that TFA counterions are toxic to cells [24,25]. It was found that peptide M33 as the TFA salt was 5–30% more toxic than the acetate salt [24]. Therefore, the use of alternative counterions, such as acetate counterions, has been considered in in vitro and in vivo studies [24].

It was reported that volatile compounds are not suitable for lyophilization since they are prone to removal using sublimation [26]. However, acetate ion, which is relatively volatile, is a commonly used salt form for several peptides including leuprolide acetate [27] and larazotide acetate [28]. While it is used for lyophilized products, there was no study found to show how to minimize the loss of volatile ions during lyophilization. The new approach to preserve volatile compounds is necessary in order to eliminate the limitation of using volatile compounds in lyophilized formulations. Moreover, the effect of the loss of counterion on the stability of protein and peptide has not been not reported.

We hypothesized that lyophilization affects the presence of the volatile counterion, which may subsequently affect the stability of the peptide, but this effect may be reduced or eliminated by choosing certain processing parameters and composition. Our current work aims to optimize excipients and processing conditions to preserve volatile counterions after lyophilization. The effect of the loss of counterions on the stability of the peptide were investigated. The stability of CSP7 as the acetate salt in both solution and lyophilized state was compared to the stability of CSP7 TFA salt in previous work. Furthermore, the stabilizing effects of different bulking agents and buffers on peptide aggregation were also studied.

## 2. Materials and Methods

### 2.1. Materials

CSP7 acetate (purity > 95%) and CSP7 trifluoroacetate (purity > 95%) were kindly provided by Lung Therapeutics Inc. (Austin, TX, USA). D-Mannitol, D-trehalose, deuterium oxide, and dimethyl sulfoxide-d_6_ (DMSO-D_6_) were purchased from Acros Organic (Morris, NJ, USA). Lactose monohydrate, sodium chloride, disodium dihydrogen phosphate, potassium dihydrogen phosphate, sodium hydroxide, ammonium hydroxide solution (28% *v/v* in water), and acetonitrile (HPLC grade) were purchased from Fisher Scientific (Fair Lawn, NJ, USA). Dulbecco’s phosphate buffered saline pH 7.4 (DPBS), which is composed of 2.7 mM potassium chloride, 1.5 mM monopotassium phosphate, 136.9 mM sodium chloride, 8.1 mM disodium phosphate heptahydrate, was purchased from Lonza (Morristown, NJ, USA). Tris(hydroxymethyl)aminomethane, hydrochloric acid solution (1.0 M), Hydranal^®^-Coulomat AG and Hydranal^®^-Coulomat CG were purchased from Sigma-Aldrich Co. (St. Louis, MO, USA). Sterile water injection USP (SWI) was purchased from Forty Aces Pharmacy (Austin, TX, USA).

### 2.2. Buffer Preparation

- The phosphate buffer solution (pH 2.0) was prepared by dissolving 50 mM disodium dihydrogen phosphate and 50 mM potassium dihydrogen phosphate in 1 L of purified water. The pH of the buffer solution was adjusted to 2.0 (±0.1) using phosphoric acid.
- The phosphate buffer solution (pH 3.5) was prepared by dissolving 50 mM potassium dihydrogen phosphate in 1 L of purified water. The pH of the buffer solution was adjusted to 3.5 (±0.1) using phosphoric acid.
- The sodium acetate buffer solution (pH 4.5) was prepared by dissolving 50 mM sodium acetate in 1 L of purified water. The pH of the buffer solution was adjusted to 4.5 (±0.1) using glacial acetic acid.
- The phosphate–citrate buffer solution (pH 5.5) was prepared by dissolving 50 mM disodium hydrogen phosphate and citric acid in 1 L of purified water. The pH of the buffer solution was adjusted to 5.5 (± 0.1) using sodium hydroxide.
- The phosphate buffer solution (pH 6.5) was prepared by dissolving 50 mM monobasic potassium phosphate in 1 L of purified water. The pH of the buffer solution was adjusted to 6.5 (±0.1) using sodium hydroxide.
- The tris-hydrochloride buffer solution (pH 7.5 and 8.0) was prepared by dissolving 50 mM tris(hydroxymethyl)aminomethane in 1 L of purified water. The pH of the buffer solutions was adjusted to 7.5 (±0.1) and 8.0 (±0.1) using hydrochloric acid.
- The tris-acetate buffer solution (pH 8.5 and 10.0) was prepared by dissolving 50 mM calcium chloride and 50 mM tris(hydroxymethyl)aminomethane in 1 L of purified water. The pH of the buffer solutions was adjusted to 8.5 (±0.1) and 10.0 (±0.1) with glacial acetic acid.
- The tris buffer saline (TBS) was prepared by dissolving 40 mM tris base and 10 mM sodium chloride in 1 L of purified water. The pH of solution was adjusted to pH 7.5 (±0.1) by adding 1 N hydrochloric acid.

### 2.3. Solubility Study

An excess amount of CSP7 peptide was added in the buffer solutions at pH 2.0, 3.5, 4.5, 5.5, 6.5, 7, 8, 8.5, and 10.0 until precipitation occurred. The samples were shaken on a vortex mixer for 5 min and on an orbital shaker at 100 rpm for 3 h. The samples were collected and centrifuged at 12,000 rpm for 15 min. The supernatants of the samples were collected and analyzed using HPLC as described in Section 2.6 below.

### 2.4. Solution Stability

The peptide solution was prepared in DPBS and TBS at 0.5 mg·ml^−1^ of free peptide. The ammonium hydroxide was added to the solution to dissolve the peptide until a clear solution was obtained.

#### 2.4.1. Storage Stability

The peptide solutions were stored at −80 °C, −20 °C, 2−8 °C, and at room temperature (25 °C). The samples were collected after 24 h and 48 h and analyzed using HPLC as described in Section 2.6 below.

#### 2.4.2. Freeze-Thaw Stability

Freeze-thaw cycles were studied at −20 °C and −80 °C. The peptide solutions were frozen at each temperature for 1 h and then thawed at room temperature (25 °C) for 1 h. The freeze-thaw cycle was repeated for 5 cycles. The samples were collected at the end of each cycle and analyzed using HPLC as described in Section 2.6 below.

#### 2.4.3. Mechanical Stability

The peptide solutions were shaken on a VWR standard orbital shaker model 1000 (Radnor, PA, USA) at 200 rpm at 5 °C and 25 °C. The samples were collected at 0.5, 1, 3, 6, and 12 h, and they were analyzed using HPLC as described in Section 2.6 below. The CSP7 aggregates were also collected and analyzed using ^1^H-NMR as described in Section 2.14 below.

### 2.5. Preparation of Lyophilized Formulations

Table 1 shows the compositions of lyophilized formulations which contained different salt forms of CSP7, bulking agents, buffers and pH modifiers. The peptide was dissolved in DPBS or TBS at a concentration of 0.5 mg·ml^−1^. A small amount of ammonium hydroxide (28% *w/w*) or 5 N of sodium hydroxide was added to adjust the pH to 8.0 (± 0.1). The peptide solution was mixed using a vortex mixer until a solution was obtained. Bulking agents was added to the solution at different molar ratios of CSP7 to bulking agent (1:5, 1:70, 1:140, 1:320) and then mixed with a vortex mixer until a clear solution was obtained. The peptide solutions were filtered through a 0.2 µm PES filter (Celltreat Scientific Products, Shirley, MA, USA). Aliquots of 2 mL of the peptide solution were filled into 5 mL borosilicate glass vials.

These glass vials were loosely closed with rubber stoppers before loading them into the lyophilizer. Samples were lyophilized using VirTis BenchTop lyophilizer (SP Scientific, Gardiner, NY, USA). The lyophilization cycle is shown in Table 2 [22]. The lyophilized chamber was filled with nitrogen gas after the secondary drying step. The vials were completely closed with rubber stoppers using compressed air inside the chamber. The vials were also sealed with an aluminum cap and stored at 2–8 °C until analysis.

### 2.6. HPLC Analysis

CSP7 was chemically analyzed with a Thermo Scientific™ Dionex™ UltiMate™ 3000 HPLC System (Thermo Scientific, Sunnyvale, CA, USA) with a wavelength of 220 nm. The two mobile phases were designated Phase A (0.1% trifluoroacetic acid in water) and Phase B (0.09% trifluoroacetic acid in a mixture of 20:80 water and acetonitrile). The HPLC gradient that operated at 1.0 mL·min^−1^ ran from 25% to 35% for mobile Phase B for 20 min then equilibrated at 25% for 5 min. An Ultimate 3000 Autosampler was utilized to inject 20 μL samples. Injections were passed through a Phenomenex^®^ Luna 5 μm C18(2) 100 Å, 150 mm × 4.6 mm (Phenomenex, Torrance, CA, USA), maintained at ambient temperature. The retention time of CSP7 was approximately 8.3 min. The standard curve of the CSP7 (concentrations of 0.015625–1.00 mg·mL^−1^) were prepared. All analyses maintained linearity in the range tested. Chromeleon Version 6.80 software (Thermo Scientific) was used to process all chromatography data.

### 2.7. Karl Fischer Titration

The moisture content of the lyophilized samples was determined using coulometric Karl Fischer titration with a C20S Compact KF Coulometer (Mettler-Toledo LCC, Columbus, OH, USA). The lyophilized powder (~30 mg) was weighed, and 1 mL anhydrous formamide was added under dry nitrogen gas in a glove bag. The solution was sonicated for 5 min or until a clear solution obtained. Solutions were withdrawn and transferred to the Karl Fischer vessel. The weight of the powder in the solution added to the vessel was calculated based on the concentration of lyophilized powder in the solution, as shown in equation (1). The moisture content of the 1 mL anhydrous formamide was also determined and subtracted from the moisture content of the samples:

(1) $$\mathrm{Weight}\;\mathrm{of}\;\mathrm{powder}\;\mathrm{added}\;\mathrm{in}\;\mathrm{the}\;\mathrm{KF}=\frac{\mathrm{Weight}\;\mathrm{of}\;\mathrm{powder}\;}{\mathrm{Weight}\;\mathrm{of}\;\mathrm{solution}}\;\times \;\mathrm{weight}\;\mathrm{of}\;\mathrm{solution}\;\mathrm{added}\;\mathrm{in}\;\mathrm{the}\;\mathrm{KF}$$

### 2.8. X-ray Diffractometry (XRD)

X-ray powder diffraction (XRD) was recorded using a benchtop X-ray diffraction instrument, model Miniflex 600 (Rigaku, Woodlands, TX, USA), with primary monochromated radiation (Cu K radiation source, λ = 1.54056 Å). The instrument was operated at an accelerating voltage of 40 kV at 15 mA. Samples were loaded in the sample holder and scanned in the range of 5–40 Å of 2θ at a scan speed of 1 °C·min^−1^, a step size of 0.04 °C·s^−1^, and a dwell time of 2 s.

### 2.9. Water Sorption Isotherms Measurement

Water sorption isotherms at 25 °C were determined for lyophilized formulations using a dynamic vapor sorption (DVS-1) microbalance (Surface Measurement Systems, London, UK). A quartz pan was filled with about 8–10 mg of powder. Samples were dried with nitrogen gas until a baseline was established less than 0.002% change in dm/dt for 120 min. Each formulation was run through a full sorption-desorption cycle from 0% to 90% RH in steps of 10% RH at 25 °C. Humidity was increased/decreased by 10% after equilibrium was reached, as determined by a dm/dt less than 0.0075% within an interval of 5 min. Sorption-desorption isotherms were calculated and plotted based on their percent change in mass compared to the initial dry formulation weight (Equation (2)):

(2) $$\%\;\mathrm{Mass}\;\mathrm{sorption}/\mathrm{desorption}\;=\left(\frac{\mathrm{Increase}\;\mathrm{or}\;\mathrm{decrease}\;\mathrm{in}\;\mathrm{mass}\;\mathrm{from}\;\mathrm{water}\;\mathrm{sorption}/\mathrm{desorption}}{\mathrm{mass}\;\mathrm{of}\;\mathrm{dry}\;\mathrm{powder}\;\mathrm{at}\;\mathrm{initial}\;\mathrm{equilibration}\;(0\%\;\mathrm{RH})}\right)\times \;100$$

### 2.10. Thermogravimetric Analysis

Thermogravimetric Analysis (TGA) was performed for ammonium acetate and ammonium TFA using a TA Thermogravimetric Analyzer Q500 (TA, Instruments, New Castle, DE, USA). Standard aluminum pans were filled with about 5–7 mg of powder. Temperature ramp experiments were performed from 30 °C to 300 °C at a rate of 5 °C·min^−1^ with a nitrogen purge at 50 mL·min^−1^.

### 2.11. Differential Scanning Calorimetry (DSC)

Differential scanning calorimetry (DSC) analysis was performed using a TA Instruments 175 Model Auto Q20 DSC. Samples of 5–7 mg were weighed and placed into standard aluminum pans. The scans were performed at a ramp rate of 5 °C·min^−1^ in the range of 30–300 °C. During the analyses, high-purity nitrogen flowed through the sample chamber at a rate of 50 mL·min^−1^.

### 2.12. Stability of Lyophilized Peptides

Formulation 7, 14, 22 and 34 were investigated for stability. Lyophilized formulations were stored in an aluminum bag with desiccators. The samples were stored at 25 °C/60% RH (for 1, 2, and 3 months) and 2–8 °C (for 1, 3, and 6 months). The samples were collected at each time point. The samples were reconstituted at 0.5 mg·mL^−1^ and investigated for aggregation after reconstitution with SWI. The recovery of CSP7 in the reconstituted solutions was analyzed by HPLC. In addition, the samples were tested for molar ratio of counterion to CSP7 and pH of reconstituted solution at each time point.

### 2.13. Stability of Lyophilized Peptides after Exposure to High Humidity Environments

Lyophilized formulations of CSP7 acetate formulated with a bulking agent (i.e., lactose, trehalose, or mannitol) buffer (DPBS/TBS) and ammonium hydroxide were prepared. Lyophilized samples were resealed and incubated at 25 °C and 75% RH for 12 h. The samples were reconstituted with SWI at 0.5 mg·mL^−1^. The reconstituted solutions were investigated for aggregation. The samples were centrifuged at 12,000 rpm for 15 min. The supernatant and aggregates were collected separately. Peptide aggregates were re-dissolved by adding a small amount of ammonium hydroxide (<1% *v/v*). The recovery of CSP7 in the supernatant and aggregates were analyzed using HPLC.

### 2.14. Solution Nuclear Magnetic Resonance

#### 2.14.1. ^1^H-NMR

We acquired ^1^H-NMR spectra using a Varian^®^ NMR 600 MHz Spectrometer (Agilent Inc., Palo Alto, CA, USA) at 25 °C. The lyophilized samples were reconstituted in deuterium oxide at 1.0 mg·mL^−1^. We added 5 μL of 1 N NaOH/D_2_O was added to the sample to dissolve the peptides. Solutions were then transferred to 5 mm NMR tubes for NMR data acquisition. Chemical shifts were referenced to a residual solvent, H_2_O, at 4.61 ppm. At this time, the ^1^H-NMR spectra were acquired using an Agilent Direct Drive 600 spectrometer, operating at a proton frequency of 599.75 MHz (14.1 T) at 298 K, equipped with an AutoX DB probe. All ^1^H-NMR spectra were recorded in D_2_O after 128 cycles for each sample. The molar ratio of counterion to peptide was calculated according to the peak area of the methyl group of CSP7 (0.85, 0.93, and 1.03 ppm; 18 H atoms in total) and the methyl group of the acetate counterion (1.75 ppm; 3 H atoms). The following equation was used to calculate molar ratio of counterion to CSP7:

(3) $$\frac{\mathrm{Molar}\;\mathrm{acetate}}{\mathrm{Molar}\;\mathrm{CSP}7}\;=\;\frac{\mathrm{peak}\;\mathrm{area}\;\mathrm{of}\;\mathrm{methy}\;\mathrm{group}\;\mathrm{of}\;\mathrm{acetate}/3}{\mathrm{peak}\;\mathrm{area}\;\mathrm{of}\;\mathrm{methyl}\;\mathrm{groups}\;\mathrm{of}\;\mathrm{CSP}7/18}$$

In addition, after the CSP7 aggregates were shaken on an orbital shaker at 25 °C for 12 h, they were collected and diluted with deuterium oxide. The ^1^H-NMR spectra were used to detect the methyl group of the acetate counterion (1.75 ppm).

#### 2.14.2. ^19^F-NMR

We acquired ^19^F-NMR spectra using a Varian^®^ NMR 600 MHz Spectrometer (Agilent Inc., Palo Alto, CA, USA) at 25 °C. The lyophilized samples were reconstituted in deuterium oxide at 1.0 mg·mL^−1^. We added 5 μL of 1 N NaOH/D_2_O to the sample to dissolve the peptide. Voriconazole was used as an internal standard. We added 50 μL of 20 mg·mL^−1^ voriconazole (VCZ)/dimethyl sulfoxide-d_6_ (DMSO-d_6_) to the sample. Solutions were then transferred to 5 mm NMR tubes for NMR data acquisition. All ^19^F NMR spectra were recorded in D_2_O after 512 cycles for each sample. The molar ratio of counterion to peptide was calculated according to the peak area of fluorine atoms of the trifluoroacetate counterion (−75.5 ppm, 3 F atoms) and the methyl group of the acetate counterion (−107.7, −110.9, and −133.5 ppm; 1F atom each). The following equations were used to calculate the molar ratio of counterions to CSP7:

(4) $$\frac{\mathrm{Molar}\;\mathrm{TFA}}{\mathrm{Molar}\;\mathrm{VCZ}}\;=\;\frac{\mathrm{peak}\;\mathrm{area}\;\mathrm{of}\;\mathrm{fluorine}\;\mathrm{of}\;\mathrm{TFA}/3}{\mathrm{average}\;\mathrm{peak}\;\mathrm{area}\;\mathrm{of}\;\mathrm{fluorine}\;\mathrm{of}\;\mathrm{VCZ}}$$

(5) $$\mathrm{Molar}\;\mathrm{of}\;\mathrm{CSP}7\;=\frac{\mathrm{weight}\;\mathrm{of}\;\mathrm{sample}\;-\;\left(\mathrm{molar}\;\mathrm{of}\;\mathrm{TFA}\;\times \;\mathrm{MW}\;\mathrm{of}\;\mathrm{TFA}\right)}{\mathrm{MW}\;\mathrm{of}\;\mathrm{CSP}7}$$

### 2.15. Molar Ratio of Counterions to CSP7

We determined the molar ratio of counterions to CSP7 in the lyophilized formulations containing different pH modifiers, buffers, and bulking agents. We achieved this using solution NMR as described in Section 2.14 above. We also investigated the molar ratios of counterions to CSP7 in lyophilized CSP7 acetate and lyophilized CSP7 TFA after 1 month and 3 months storage at 25 °C/60% RH and after 6 months storage at 5 °C. Additionally, the molar ratios of acetate to CSP7 were determined in lyophilized formulations containing different concentrations of acetic acid (7%, 14%, and 21% *v/v*). Furthermore, the molar ratio of acetate to CSP7 in CSP7 acetate unprocessed powder was determined after exposure to the air and exposure to different humidity including 30% RH, 50% RH, 70% RH, and 90% RH.

### 2.16. Statistical Analysis

For statistical analysis, we tested the solubility of CSP7 acetate and TFA at different pH levels of the aggregates in Formulations after storage at 25 °C and 60% RH for 1 month and 3 months. We also tested the molar ratios of acetate to CSP7 in the lyophilized formulations at the initial point and then after storage at 25 °C and 60% RH and then at 5 °C. All statistical analyses were tested using the student’s *t*-test (*p*-value < 0.05) and were reported.

## 3. Results

### 3.1. Comparison of Physicochemical Properties of CSP7 Acetate and CSP7 TFA

#### 3.1.1. Solubility of CSP7

**Figure 1:**
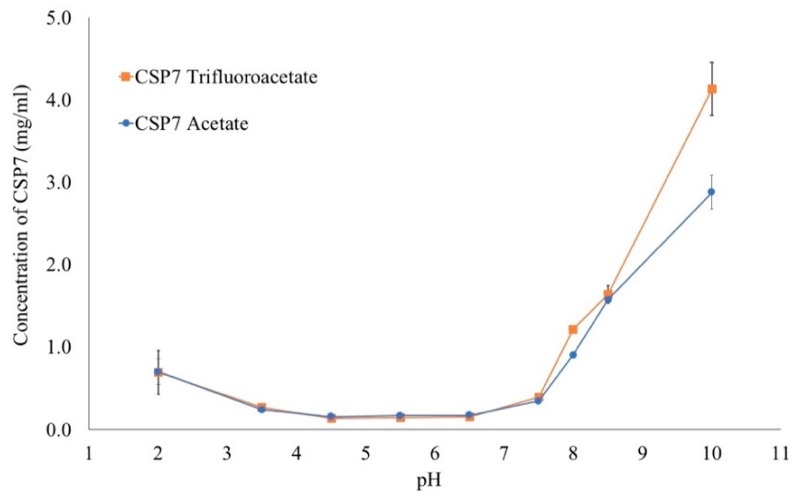
pH solubility profile of CSP7 acetate and CSP7 TFA.

The solubility of CSP7 acetate and CSP7 TFA was determined between pH 2 and pH 10, and is shown in Figure 1. The solubility of CSP7 acetate at pH 2 was 0.701 ± 0.153 mg·mL^−1^ and it continuously decreased to 0.241 ± 0.007 mg·mL^−1^ (*p* < 0.05) at pH 3.5, and 0.160 ± 0.003 mg·mL^−1^ (*p* < 0.05) at pH 4.5 as the pH increases. Between pH 4.5 and 6.5, the solubility of CSP7 acetate increased slightly, and it was 0.174 ± 0.016 mg·mL^−1^ at pH 5.5, and 0.178 ± 0.013 mg·mL^−1^ at pH 6.5. As pH was increased, the solubility of CSP7 acetate increased to 0.347 ± 0.014 mg·mL^−1^ at pH 7.5, and it increased between pH 7.5 and pH 10.0 (2.880 ± 0.201 mg·mL^−1^).

The pH-solubility profile of CSP7 TFA was similar to that of CSP7 acetate. The solubility of CSP7 TFA was not found to be significantly different that of CSP7 acetate (*p* > 0.05) at pH 2.0, 3.5, 4.5, 5.5, 6.5, 7.5, and 8.5. However, the solubility of CSP7 TFA at pH 8.0 and 10.0 was significantly higher than CSP7 acetate (*p* < 0.05). The solubility of CSP7 TFA at pH 8.0 and 10.0 was 1.216 ± 0.024 and 4.134 ± 0.320 mg·mL^−1^, respectively.

#### 3.1.2. Moisture Sorption-Desorption Isotherm of CSP7 Acetate and CSP7 TFA

**Figure 2:**
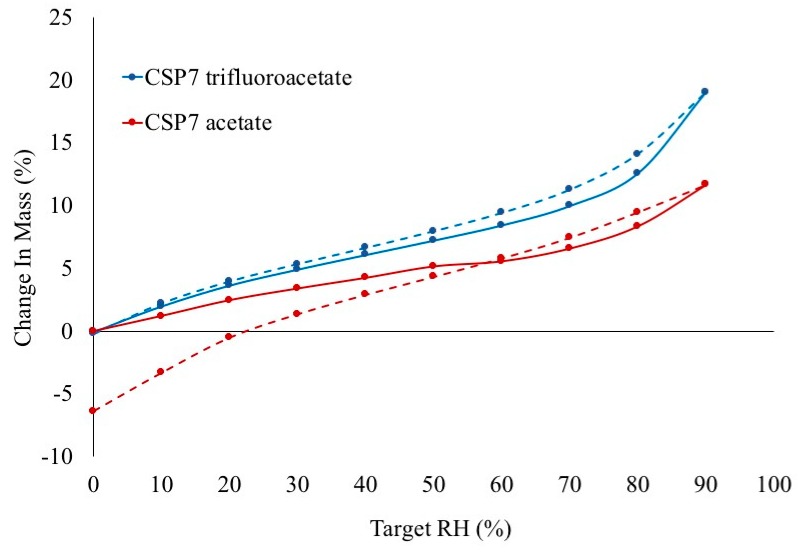
Moisture sorption (solid line) and desorption (dotted line) isotherms of CSP7 acetate and CSP7 TFA.

The moisture sorption of different salt forms of CSP7 were compared. Figure 2 shows the water sorption-desorption isotherm of CSP7 acetate and CSP7 TFA. CSP7 acetate and CSP7 TFA were crystalline in unprocessed powder form. Crystalline materials are less prone to absorb water since they can only adsorb water at the surface, while amorphous materials can also absorb water at the surface and allow water to diffuse through the material [29]. The moisture sorption isotherm of CSP7 acetate showed a decrease in mass change at 60% RH. This indicates that the small amount of amorphous in CSP7 acetate recrystallized and exhibited less moisture sorption after recrystallization at 60% RH. In contrast, CSP7 TFA exhibited constant bulk material weight during both sorption and desorption, which confirms that no change occurred in the physical state of CSP7 TFA after exposure to 75% RH. Additionally, the moisture desorption isotherm of CSP7 acetate showed a 6.36% decrease in weight compared to its initial weight, while the moisture desorption isotherm of CSP7 TFA showed no change in weight. These results indicate that moisture induced the loss of mass from the CSP7 acetate bulk powder.

#### 3.1.3. Counterion Volatilization

**Figure 3:**
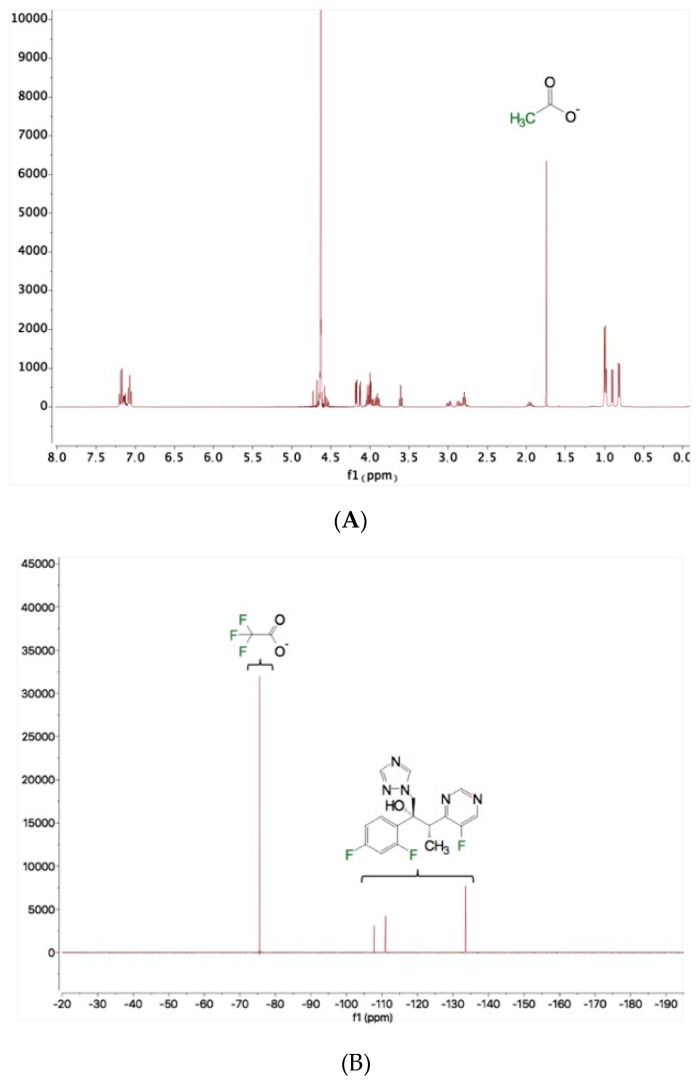
NMR spectrums of bulk powder: (**A**) ^1^H-NMR spectrum of CSP7 acetate; (**B**) ^19^F-NMR spectrum of CSP7 TFA.

**Figure 4:**
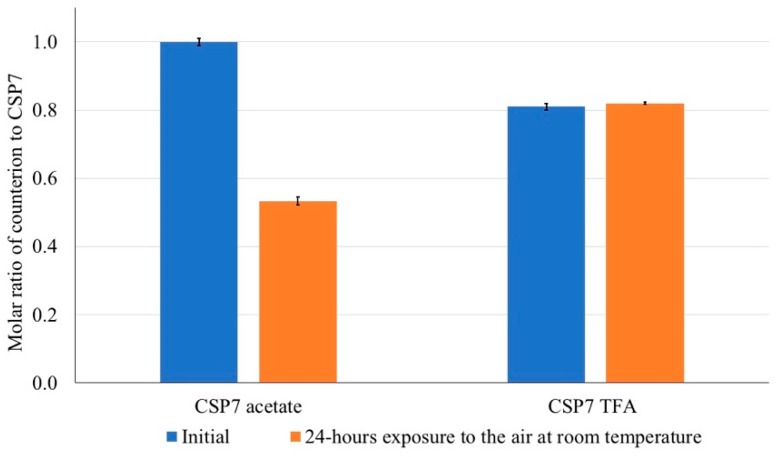
Molar ratio of acetate to CSP7 after exposure to air at room temperature.

The presence of counterions in the bulk material and powder after exposure to air was analyzed using solution NMR. As shown in Figure 3A, the peak of acetate was observed at 1.75 ppm in the ^1^H- NMR spectrum. The peak of trifluoromethyl (CF_3_) was observed at −75 ppm in the ^19^F-NMR spectrum (Figure 3B). The molar ratio of counterions to CSP7 was calculated as described in Section 2.15 above. In the bulk material, the molar ratio of acetate to CSP7 was 1:1, while the molar ratio of TFA to CSP7 was 0.81:1 (Figure 4). After 24 h of exposure to air at room temperature, the molar ratios of acetate to CSP7 in the bulk CSP7 acetate was 0.53:1, while the molar ratio of TFA to CSP7 in the bulk CSP7 TFA remained 0.82:1 after 24 h exposure to air at room temperature.

**Figure 5:**
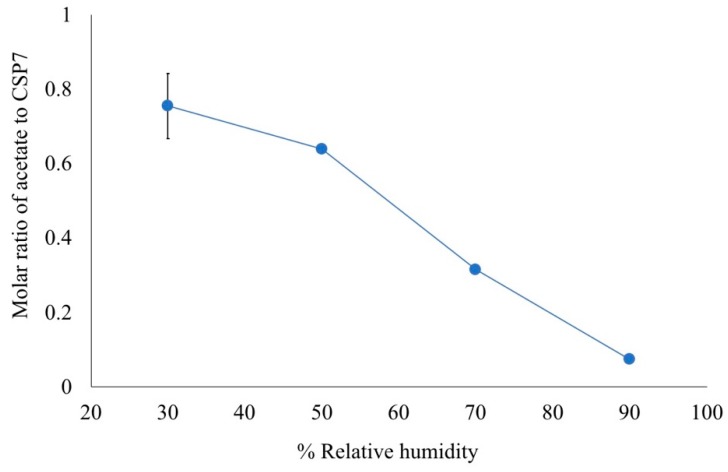
Molar ratio of acetate to CSP7 in bulk material after exposure to different humidity levels.

The effect of moisture on the volatilization of acetate counterions was investigated after they were exposed to moisture at different levels of humidity. Figure 5 shows the molar ratios of acetate to CSP7 in the samples after incubation. The molar ratios of acetate to CSP7 after incubation at 30, 50, 70, and 90% RH were 0.75:1, 0.64:1, 0.3:1, and 0.07:1, respectively.

#### 3.1.4. Stability of CSP7 Acetate in Solution

The stress conditions (i.e., mechanical agitation, freeze–thaw cycles, storage at different temperatures) were selected to evaluate the stability of peptides in different buffers including DPBS and TBS. Table 3 shows the effect of storage temperature on the stability of CSP7 acetate. The solutions were clear after storage for up to 48 h. The recovery of CSP7 in DPBS and TBS was about 100% after 48 h storage at −80 °C, −20 °C, 5 °C, and 25 °C (Table 3). These results indicate that storage temperatures between −80 °C and 25 °C did not affect the stability of CSP7 acetate for up to 48 h. Table 4 shows the effect of freeze-thaw cycles. The CSP7 in DPBS and TBS was physically and chemically stable after multiple freeze-thaw cycles at −80 °C and −20 °C. Peptide aggregation or precipitate was not detected in any samples after multiple freeze-thaw cycles. The CSP7 remained about 100% in DPBS and TBS after five freeze-thaw cycles at −80 °C and −20 °C, respectively.

**Figure 6:**
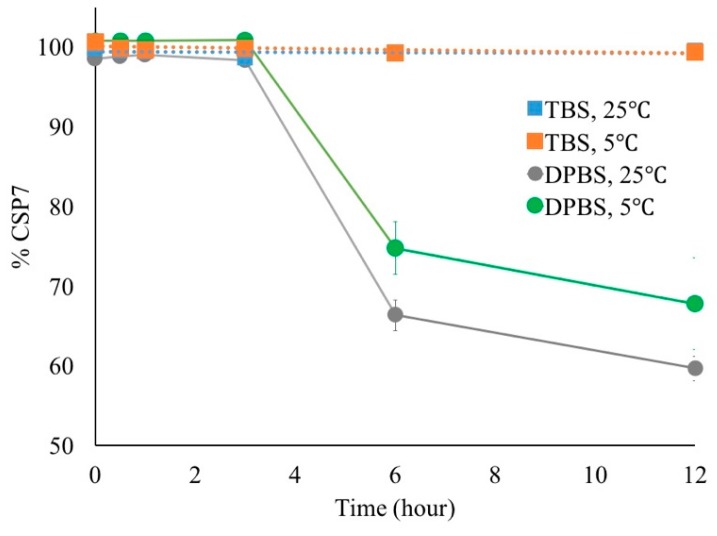
Recovery of CSP7 acetate in bulk solution after mechanical shaking.

**Figure 7:**
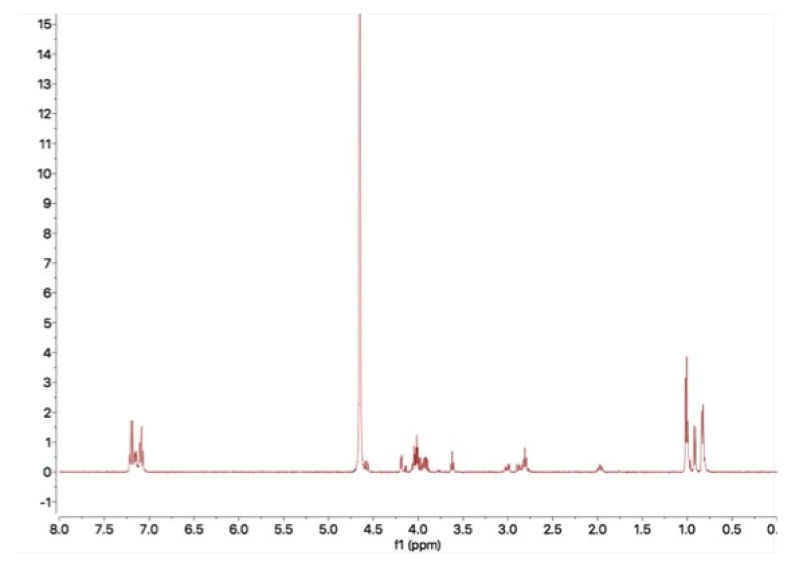
^1^H-NMR spectrum of CSP7 aggregates after 12 h shaking on an orbital shaker at 25 °C.

Figure 6 shows the effect of mechanical agitation on the recovery of CSP7 in DPBS and TBS. The agitation affected the physical stability of CSP7 acetate in DPBS at both 5 °C and 25 °C. Peptide aggregation was found after 6 h for shaking on the orbital shaker. The recovery of CSP7 in the supernatant was 67.8 ± 5.8% and 59.7 ± 1.5% after 12 h of shaking at 5 °C and 25 °C, respectively. The recovery of CSP7 in the aggregates was 31.7 ± 2.3% and 38.3 ± 2.8% after 12 h of shaking at 5 °C and 25 °C, respectively. In contrast, the solutions containing TBS remained clear and colorless for 12 h while shaken on the orbital shaker. No peptide aggregation or precipitation was detected in any samples after shaking. The CSP7 acetate potency remained at 99.4% and 99.5% after 12 h shaking on an orbital shaker at 5 °C and 25 °C, respectively. Additionally, aggregates were collected after shaking at 25 °C for 12 h and then analyzed by ^1^H-NMR. The NMR spectrum showed no peak for acetate counterions (Figure 7), which indicates that the soluble aggregates were formed by free peptide molecules.

### 3.2. Stabilizing Effect of Bulking Agents against Moisture

We studied how the choice of bulking agent affects the stability of CSP7 acetate in the presence of moisture. We optimized the molar ratio of CSP7 to the bulking agent, the types of bulking agents, and buffers. The content of the bulking agent affected product appearance, the osmolality of the reconstituted solutions, the residual moisture content, and the recovery of CSP7 after lyophilization.

Among all three bulking agents, mannitol produced elegant, smooth, and homogeneous cakes, while lactose and trehalose produced shrinkage and slightly shrunken cakes. The cake appearance was related to the residual moisture content in the lyophilized powder. The moisture content of lyophilized mannitol-based formulations was 1–4.5%, while formulations based on lactose and trehalose had a moisture content of 1.5–8.1%.

The residual moisture content of the lactose- and trehalose-based formulations were higher than the mannitol-based formulations; however, all reconstituted solutions were clear. The recovery of CSP7 after lyophilization was more than 96.5%. In contrast, CSP7 in some mannitol-based formulations were not stable after lyophilization. The reconstituted solutions of formulations 8, 9, 10, and 11 were turbid. Soluble aggregates were found in formulations 8, 9, 10, and 11. The recovery of CSP7 in the supernatant was 69.4 ± 4.2, 86.3 ± 1.8, 40.1 ± 0.7, and 64.1 ± 0.5 respectively, for these formulations. Other formulations were stable after lyophilization. The recovery of CSP7 was more than 96%.

To investigate the stabilizing effect of bulking agents in different buffer systems, the lyophilized formulations were exposed to 25 °C/75% RH for 12 h. Turbid solutions were found after the lyophilized cakes were reconstituted with SWI. The peptide aggregates were collected and added into the buffer. pH of DPBS and TBS was 7.4 ± 0.1 and 7.6 ± 0.1, respectively. A small amount of ammonium hydroxide (less than 1% *v/v*) was added into the buffer to increase pH and subsequently enhance the solubility of CSP7. The aggregates can be re-dissolved after adding ammonium hydroxide and adjust pH to 8.0 ± 0.1. CSP7 multimers and degradation products were not found in any CSP7 aggregates using HPLC. These results confirm that the soluble aggregates were reversible and converted to a soluble monomeric species of the CSP7 peptide.

**Figure 8:**
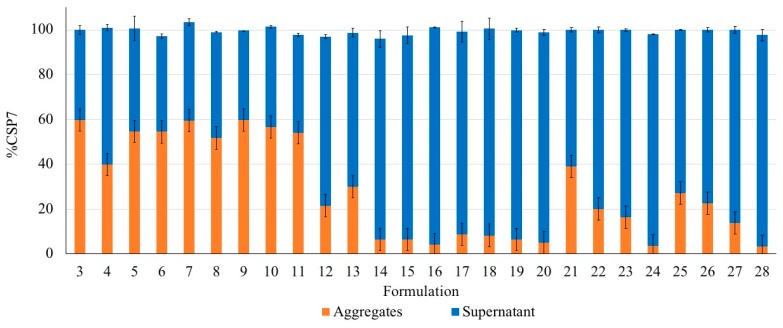
Recovery of CSP7 acetate in soluble aggregates and supernatant after exposure of the lyophilized CSP7 acetate formulations to 25 °C and 75% RH for 12 h.

Figure 8 shows the amount of CSP7 in the aggregates and the supernatant. The amount of aggregate in the formulations based on mannitol, lactose, or trehalose was in the range of 14.6–59.8%, 4.3–29.9%, and 3.5–37.0%, respectively. The mannitol-based formulations showed significantly more aggregates than the lactose- and trehalose-based formulations when compared at the same molar ratio. The content of lactose and trehalose affected peptide aggregation. As the amount of lactose or trehalose increased from a 1:5 molar ratio to 1:140, fewer peptide aggregates were observed. In contrast, the aggregate content of mannitol-based formulations at different molar ratios were slightly different.

### 3.3. Physical State of Lyophilized Formulations

**Figure 9:**
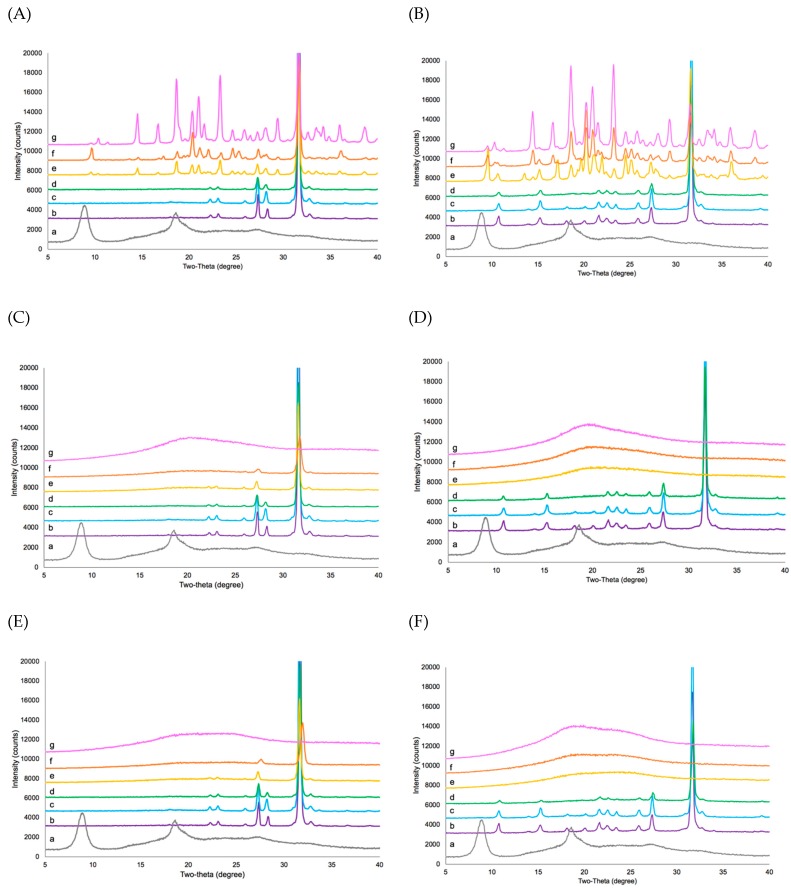
X-ray diffraction patterns: (**A**) lyophilized mannitol (DPBS) formulations; (**B**) lyophilized mannitol (TBS) formulations; (**C**) lyophilized lactose (DPBS) formulations; (**D**) lyophilized lactose (TBS) formulations; (**E**) lyophilized trehalose (DPBS) formulations; (**F**) lyophilized trehalose (TBS) formulations. [a: CSP7 acetate, b: lyophilized pure buffer, c: lyophilized neat CSP7, d: lyophilized 1:5-CSP7:bulking agent, e: lyophilized 1:70-CSP7:bulking agent, f: lyophilized 1:140-CSP7:bulking agent, g: lyophilized 1:320-CSP7:bulking agent].

Figure 9 shows the XRD diffractograms of lyophilized formulations. The sharp peaks at 26.0, 27.5, and 31.5 degrees 2θ that were present in most of the lyophilized formulations indicated crystal peaks of sodium chloride from the buffers. In DPBS-based formulations, the sharp peaks at 22.5, 23.0, and 28.5 degrees 2θ were indicated crystal peaks of phosphate salt from the buffer. As the content of the bulking agent increases, the small peaks were absent, since they were not present in the XRD diffractograms of lyophilized 1:320-CSP7 acetate:bulking agent. In the TBS-based formulation, the sharp peaks at ~10.5, 15.0, 18.0, 20.0, 22.5, and 23.5 degrees 2θ indicate crystal peaks of TRIS. Similar to DPBS-based formulations, the small peaks did not present in lyophilized 1:70, 1:140 and 1:320-CSP7 acetate:bulking agent.

The physical state of the bulking agents after lyophilization was also analyzed using XRD. Bulk mannitol powder (before processing) was in the β crystalline form, which exhibits sharp peaks at 14.5, 16.8, 18.8, and 23.7 degrees 2θ. The lyophilized 1:5-CSP7 acetate:mannitol formulation that included DPBS or TBS was amorphous.

Figure 9A,B show that lyophilized 1:70-CSP7 acetate:mannitol, 1:140-CSP7 acetate:mannitol, and 1:320-CSP7 acetate:mannitol formulations that included DPBS or TBS contained a mixture of β, α, and δ crystalline forms [30]. As the amount of mannitol was increased, the intensity of the crystalline mannitol increased. Bulk lactose unprocessed powder was in α-lactose monohydrate form. There was no peak of α-lactose monohydrate (i.e., peaks at 12.5, 16.4, and 20.0 degrees 2θ) [31] in any of the lyophilized CSP7-lactose molar ratios formulated with DPBS or TBS (Figure 9C,D). Similar to CSP7-lactose formulations, there was no peak representing d-trehalose in all lyophilized CSP7-trehalose molar ratios in formulations containing DPBS or TBS (Figure 9E,F). These results indicate that lactose and trehalose were amorphous after lyophilization.

### 3.4. The Presence of Counterions after Lyophilization

#### 3.4.1. Effect of Lyophilization on the Loss of Counterions

**Figure 10:**
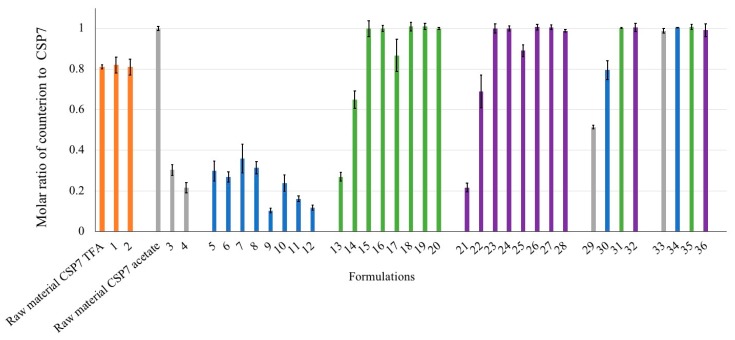
Molar ratio of counterions to CSP7.

We analyzed the molar ratio of counterions to CSP7 in lyophilized CSP7 acetate and CSP7 TFA to confirm whether counterions remained in the samples after lyophilization. The molar ratio of acetate to CSP7 in lyophilized CSP7 in DPBS without a bulking agent was 0.30:1 (Figure 10, formulations 3 and 4). This result indicates the loss of acetate after lyophilization. In contrast to CSP7 acetate, the amounts of TFA in the CSP7 TFA bulk powder and in the lyophilized samples were not significantly different. The molar ratios of TFA to CSP7 in the bulk raw material and in formulation 1 were 0.81:1 and 0.81:1, respectively (Figure 10). These results indicate that TFA counterions were not lost after lyophilization.

#### 3.4.2. Effect of Excipients on the Preservation of Counterions in Lyophilized Compositions

We also studied the effect of excipients on the presence of counterions. In the lyophilized CSP7-lactose and CSP7-trehalose formulations, the molar ratio of acetate to CSP7 significantly increased as the content of lactose or trehalose increased (*p* < 0.05). The effect of buffers on the presence of counterions was also found in lactose- and trehalose-based formulations. The molar ratios of acetate to CSP7 at 1:5-CSP7 acetate:lactose with the combination of DPBS and TBS were 0.27:1 and 0.87:1, respectively (Figure 10, formulations 13 and 17). Similarly, the molar ratios of acetate to CSP7 at 1:5-CSP7 acetate:trehalose with the combination of DPBS and TBS were 0.22:1 and 0.89:1, respectively (Figure 10, formulations 21 and 25).

These results indicate that acetate ions remained in higher amounts in TBS-based formulations, as compared to DPBS-based formulations. However, these trends contradict the results observed in the mannitol-based formulations. The molar ratios of acetate to CSP7 were in the range of 0.20:1 to 0.36:1 (Figure 10, formulations 5–12). These results show no correlation between the content of the remaining acetate ions and the content of mannitol in the lyophilized formulations.

In addition to buffers and bulking agents, we investigated the effect of pH modifiers used to increase the solubility of CSP7 in lyophilized formulations. The use of ammonium hydroxide resulted in lower molar ratios of acetate to CSP7 in the lyophilized samples, while the use of sodium hydroxide did not affect the loss of counterions. The molar ratio of acetate to CSP7 in the lyophilized samples containing sodium hydroxide, and without bulking agent, remained 1:1 (Figure 10, formulation 33).

**Figure 11:**
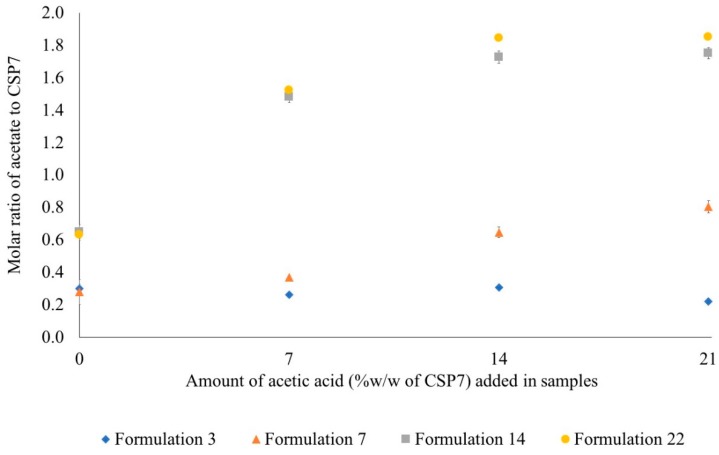
Molar ratio of counterions to CSP7 in lyophilized formulations containing varying amounts of acetic acid.

We also compared the capabilities of various bulking agents to preserve acetate counterions after lyophilization of CSP7 acetate with the addition of acetic acid. The molar ratio of acetate to CSP7 in the CSP7 acetate bulk powder was about 1:1, which was equivalent to 7% *w/w* of bulk powder. We added 7%, 14%, and 21% *v/v* of acetic acid in CSP7 acetate solutions and then lyophilized them with a combination of bulking agent and DPBS. As shown in Figure 11, higher molar ratios of acetate to CSP7 were shown in lyophilized samples containing higher concentrations of acetic acid. The molar ratios of acetate to CSP7 in formulation 14 containing 7%, 14%, and 21% *w/w* acetic acid were 1.48:1, 1.73:1, and 1.75:1, respectively. The molar ratios of acetate to CSP7 formulation 22 containing 7%, 14%, and 21% *w/w* acetic acid were 1.52:1, 1.84:1, and 1.85:1, respectively. The molar ratios of acetate to CSP7 in formulation 7 containing 7%, 14%, and 21% *v/v* acetic acid were 0.37:1, 0.65:1, and 0.80:1, respectively.

#### 3.4.3. Effect of Vacuum Pressure on the Preservation of Counterions in Lyophilized Formulations

We investigated the effect of vacuum pressure on the sublimation rate of acetate counterions during freeze drying. The lyophilized formulations containing ammonium hydroxide were prepared at a higher vacuum pressure during the drying steps. The appearance of the lyophilized cakes prepared at 350 mTorr were similar to that of the lyophilized cakes prepared at 100 mTorr. The molar ratios of acetate to CSP7 in the lyophilized neat CSP7 acetate prepared at 100 and 350 mTorr were 0.30:1 and 0.51:1, respectively (Figure 10, formulations 3 and 30). Additionally, the lyophilized CSP7 acetate with bulking agents showed the same trend. The molar ratios of acetate to CSP7 in lyophilized 1:140-CSP7 acetate:mannitol with the combination of DPBS and ammonium hydroxide prepared at 100 and 350 mTorr were 0.36:1 and 0.79:1, respectively (Figure 10, formulations 7 and 34). Accordingly, the molar ratios of acetate to CSP7 in formulations 31 and 32 prepared at 350 mTorr were all approximately 1:1, which is higher than the molar ratio of acetate to CSP7 in the formulations contained same compositions but prepared at 100 mTorr (formulation 14 and formulation 22, respectively).

### 3.5. Stability of Lyophilized Formulations

**Figure 12:**
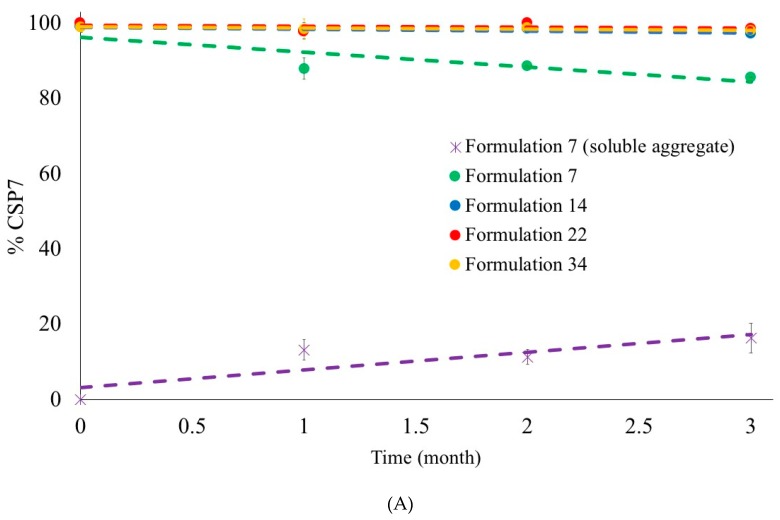

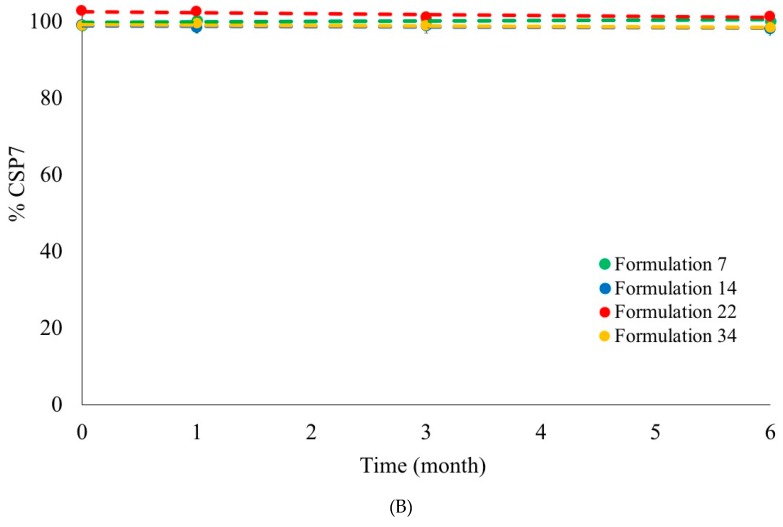
Recovery of CSP7 in lyophilized state after storage: (**A**) 25 °C/60% RH; (**B**) 5 °C.

The chemical and physical stability of formulations 7, 14, 22, and 34 were compared, and the results are presented in Figure 12. The stability of lyophilized CSP7 acetate was investigated in sealed vials under dry nitrogen gas and stored at 25 °C/60% RH and 5 °C. Formulations 14, 22, and 34 were chemically and physically stable (>97%) after storage at 5 °C for six months and at 25 °C for three months. Formulation 7 was stable at 5 °C for up to six months (>96%) but was physically unstable after storage at 25 °C/60% RH for one month. Soluble aggregates were found after one month of storage at 25 °C/60% RH. The recovery of CSP7 in the supernatant and aggregates was 86.4% (±3.3%) and 14.5% (±0.8%), respectively. After three months of storage at 25 °C/60% RH, the recovery of CSP7 in the supernatant and aggregates was 84.5% (±2.05%) and 16.3% (±4.01%), respectively. The level of peptide aggregates in the formulations after one month of storage at 25 °C/60% RH were not significantly different from those of samples after three months of storage at 25 °C and 60% RH.

Table 5 shows the pH levels of the reconstituted solutions, which was measured immediately after reconstitution. The pH of the reconstituted solution of Formulation 7 was 7.60 (±0.02), while the pH of the reconstituted solutions of Formulations 22 and 14 were 7.81 (±0.02) and 7.84 (±0.05), respectively. Additionally, the pH of the reconstituted solutions of Formulation 34 was 8.06 (±0.04), which was significantly higher than that of Formulation 3 (*p* < 0.05).

The pH of the reconstituted solutions of all lyophilized formulations did not change after storage at 5 °C up to six months. After storage at 25 °C/60% RH, the pH of the reconstituted solutions of Formulations 14, 22, and 34 did not significantly change after storage up to three months. However, the pH of the reconstituted solutions of Formulation 7 showed a significant decrease in the pH of the reconstituted solutions after storage at 25 °C/60% RH (*p* < 0.05). For lyophilized CSP7 TFA, the pH of the reconstituted solution of Formulation 34 was slightly higher than that of Formulation 7, and the pH level did not significantly change after storage at both 5 °C and 25 °C/60% RH.

### 3.6. Molar Ratios of Counterions to CSP7 in Lyophilized Formulations after Storage

**Figure 13:**
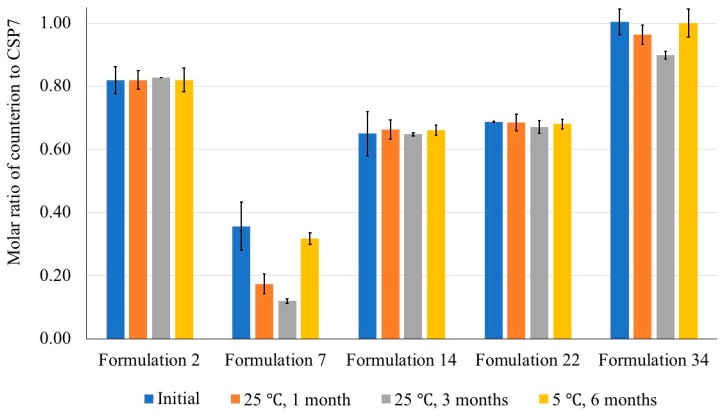
Molar ratio of counterions to CSP7 after storage.

We investigated the molar ratios of counterions to CSP7 in lyophilized samples after storage. As shown in Figure 13, the molar ratios of acetate to CSP7 in all formulations (nos. 1–5) did not change after storage at 5 °C for six months. For lactose- and trehalose-based formulations, there was no significant difference in the molar ratios of acetate to CSP7 in Formulations 14 and 22 after storage at 25 °C/60% RH up to three months. However, the molar ratios of acetate to CSP7 in the mannitol-based formulations (Formulations 7 and 34) significantly decreased after one month of storage at 25 °C/60% RH. The molar ratio of acetate to CSP7 in Formulation 34 decreased slightly from 1:1 to 0.96:1 after one month of storage at 25 °C/60% RH. The molar ratio of acetate to CSP7 in Formulation 7 decreased significantly from 0.36:1 to 0.17:1 after one month of storage at 25 °C/60% RH (*p* < 0.05). In addition to CSP7 acetate, the molar ratio of TFA to CSP7 in lyophilized 1:140-CSP7 TFA-mannitol combined with DPBS and ammonium hydroxide (i.e., Formulation 2) did not significantly change after one month of storage.

### 3.7. Thermal Analysis of Ammonium Acetate and Ammonium TFA

**Figure 14:**
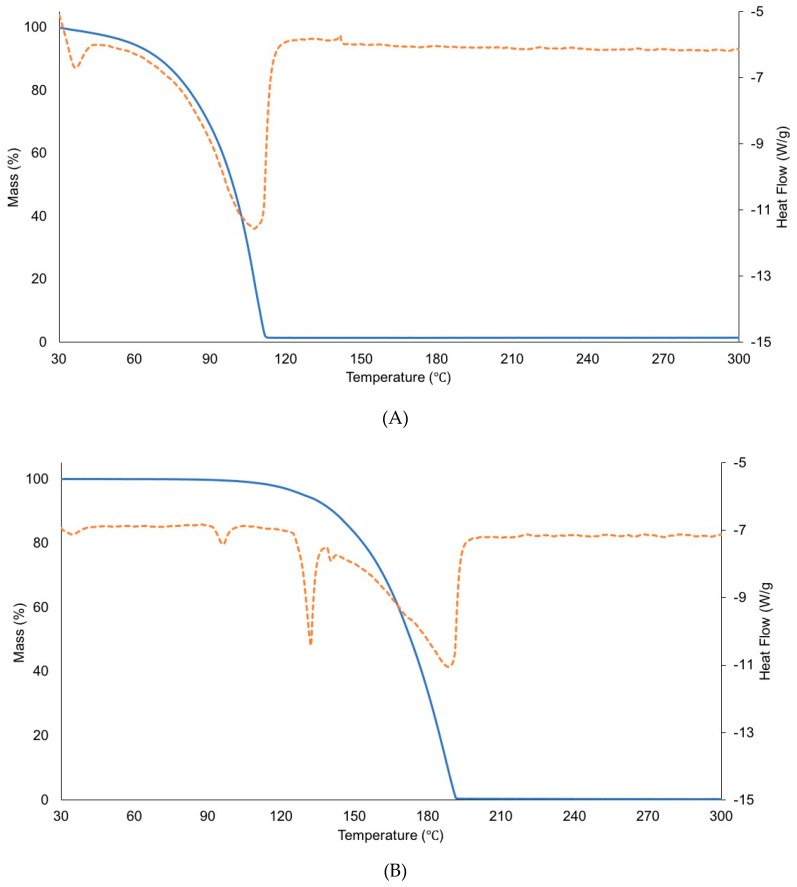
TGA data (solid line) overlaid with DSC analysis data (dotted line): (**A**) ammonium acetate; (**B**) ammonium TFA.

We used thermogravimetric analysis (TGA) and differential scanning calorimetry (DSC) analysis to study the melting and boiling points of ammonium acetate and ammonium TFA. Figure 14 presents data from TGA overlaid with data from DSC. The TGA data reveal that ammonium acetate experienced a continuous mass loss after the sample was heated from 30 °C (see Figure 14A). Then, the mass of ammonium acetate dramatically decreased to less than 1% after it was heated to ~110 °C (see Figure 14A). No sample remained in the pans after analysis. The DSC data of ammonium acetate demonstrate that both melting and boiling occurred simultaneously, and the data show the overlapped peaks of melting and boiling. For ammonium TFA, the TGA and DSC data show that the mass of ammonium TFA decreased after melting at ~130 °C and decreased to 0% after boiling at ~190 °C (see Figure 14B). These results indicate that ammonium acetate easily evaporated after it changed to its liquid phase, since it showed a substantial loss of ammonium acetate during melting.

## 4. Discussion

### 4.1. Various Counterions of CSP7 Affect the Physicochemical Properties of CSP7

These results demonstrate that various counterions of CSP7 affect the physicochemical properties of CSP7. Several papers reported that counterion has an impact on solubility, stability and solid-state properties of compounds [20,32]. In our case, we found various counterions of CSP7 affect the solubility of CSP7 especially in basic conditions. Interestingly, the results also point out that the counterion also affects counterion volatilization. NMR results show that only acetate counterions were lost significantly after 24 h exposure to air at room temperature, while TFA counterions remained unchanged. This result corresponds with moisture sorption thermograms. The loss of mass of CSP7 acetate bulk powder agrees with the molar ratio of acetate to CSP7 in bulk CSP7 acetate after exposure to 90% RH. We used the molar ratios of acetate to CSP7 (obtained from NMR) in the bulk CSP7 acetate in its initial state and after exposure to calculate the percentage of mass loss (% *w/w*) (Equation (6)). The percentage of mass loss after exposure was 6.37%, which agrees with the percentage of mass loss shown in the moisture sorption thermogram. Both the NMR and DVS results indicate that acetate counterions are more volatile than TFA counterions.

(6) $$\%\;\mathrm{Mass}\;\mathrm{loss}\;=\;\frac{{\left(\mathrm{molar}\;\mathrm{of}\;\mathrm{acetate}\;\times \;\mathrm{MW}\;\mathrm{of}\;\mathrm{acetate}\right)}_{\mathrm{initial}}\;-\;\left(\mathrm{molar}\;\mathrm{ratio}\;\mathrm{of}\;\mathrm{acetate}\;\times \;\mathrm{MW}\;\mathrm{of}\;\mathrm{acetate}\right){\;}_{\mathrm{after}\;\mathrm{incubation}}}{{\left(\mathrm{molar}\;\mathrm{of}\;\mathrm{acetate}\;\times \;\mathrm{MW}\;\mathrm{of}\;\mathrm{acetate}\right)}_{\mathrm{initial}}\;+\;\left(\mathrm{molar}\;\mathrm{ratio}\;\mathrm{of}\;\mathrm{CSP}7\;\times \;\mathrm{MX}\;\mathrm{of}\;\mathrm{CSP}7\right){\;}_{\mathrm{initial}}}$$

Various counterions also affect the stability of CSP7 in solution. Hengsawas et al. previously reported that CSP7 TFA was stable after 24 h of shaking on an orbital shaker at 5 °C and 25 °C [22]. However, we found that CSP7 acetate was also physically unstable after 6 h of shaking on an orbital shaker at 5 °C and 25 °C. The NMR spectrum of CSP7 aggregates confirms that acetate counterions dissociate from CSP7, leaving free peptide molecules to interact with one other and subsequently form aggregates. It has been reported that electrostatic interactions play important roles in the self-association of peptides to form aggregates [33]. Marek et al. showed that the lower the net change, the higher the propensity to aggregate [34]. Therefore, cations and anions in solution affect both the rate and the extent of aggregation [34].

We hypothesize that the interaction between TFA and CSP7 in solution is stronger than the interaction between acetate and CSP7. TFA is composed of three fluorine atoms, which are more hydrophobic than the three hydrogen atoms of the acetate ions. It is possible that the TFA^−^ anion can exhibit either (a) an ionic interaction with the *N*-terminal of the peptide or (b) a hydrophobic interaction between the fluorine atoms in TFA and the phenyl groups of the amino acid residues. The interaction between ions and peptide possibly helps minimize the self-association of peptide molecules, thus minimizing the formation of aggregates.

Moreover, it was reported that ions affect the solubility and stability of peptide and proteins through salting out [35]. The mechanism of the effect of ions on the stability of proteins and peptides is currently unclear, but it was reported that protein salting out capability for salts are dependent on the ion hydration properties [36]. Large and more polarizable anions are less hydrated than the small and less polarizable anions [36]. Compared with TFA, the acetate ions, smaller and less polarizable anions, are reported as strongly hydrated ions [36]. Therefore, it seems possible that acetate ions tend to have a strong interaction with water, thus reducing the amount of water surrounding the peptides. Therefore, peptide solubility decreases.

### 4.2. Effect of the Loss of Counterions on the Stability of CSP7

We found that lyophilization affects the presence of acetate counterions. Since we found no study reporting whether the loss of counterion affect the stability of peptide, the stability of lyophilized formulations containing different molar ratios of acetate to CSP7 was investigated. The results show that Formulation 7, which contained a low molar ratio of acetate to CSP7, was physically unstable after one month of storage at 25 °C/60% RH. On the other hand, Formulation 34, which contained a high molar ratio of acetate to CSP7, was stable after storage at 25 °C/60% RH up to three months (Figure 12). Interestingly, we also found that the molar ratio of acetate to CSP7 in Formulation 7 decreased in larger amounts during storage at 25 °C/60% RH compared to Formulation 34 (Figure 13).

The effect of various counterions on the stability of CSP7 was also compared with the results from the previous study. Hengsawas et al. reported that Formulation 5 was chemically and physically stable after one month of storage at 25 °C and 60% RH [22]. We found that lyophilization did not affect the presence of TFA counterions. ^19^F NMR data demonstrate that the molar ratio of TFA to CSP7 in Formulation 2 was constant after three months of storage under the same conditions (Figure 10). This indicates that the difference in stability between lyophilized CSP7 acetate and CSP7 TFA is associated with the presence of counterions.

When comparing Formulations 7 and 34, it is clear that pH modifiers play a substantial role in the loss of counterions. CSP7 acetate was dissolved in DPBS or TBS, and its pH was adjusted to 8.0. After pH adjustment, it was postulated that the pH modifier could interact with the free counterions that dissociated from the CSP7 salt. Ammonium hydroxide can interact with free acetate ions or free trifluoroacetate ions and subsequently form ammonium acetate or ammonium TFA Similarly, sodium hydroxide can interact with free acetate ions and subsequently form sodium acetate.

Consistent with the literature, the salt formation of the drug and excipients can occur during lyophilization [37]. The various salts that form during preparation have different physicochemical properties, which affects the physicochemical properties of lyophilized formulations [37]. The boiling points of ammonium acetate and sodium acetate were 138.5 °C [38] and 881.4 °C [39], respectively. Ammonium acetate is a volatile salt with a high vapor pressure (13.9 mm Hg, at 25 °C) [40].

As shown in Figure 14, the mass of ammonium acetate decreased significantly at lower temperatures compared to ammonium TFA. This indicates that the ammonium acetate was more volatile than the ammonium TFA. Based on the boiling point, it seems plausible that the ammonium acetate could be sublimated at low pressure during the drying step of the process, while ammonium TFA and sodium acetate could remain in lyophilized samples. This agrees with the literature that shows that volatile compounds such as ammonium formate, acetate, or bicarbonate are easily removed during the ice sublimation stage, and they do not remain in the lyophilized product [26,41].

Interestingly, we found that the level of acetate counterions in lyophilized CSP7-mannitol was also decreased during storage at 25 °C/60% RH, while the level of TFA counterions remained the same during storage under the same conditions. Moisture is one factor that affects the loss of counterions. As the moisture was increased, the molar ratio of acetate to CSP7 decreased (Figure 5). Volatilization of the counterions after exposure to high humidity was also reported by Thakral. The volatilization of HCl counterions after the disproportionation of the HCl salt and HBr salt occurred during accelerated stability studies (at 40 °C/75% RH) [42]. Moisture can accelerate the disproportionation of the salts, which causes a drop in the pH of the formulation. Consequently, the dissociated HBr counterion from the salt in the mobile water caused a decrease in the dissolution of formulations. This agrees with our observation.

The volatility of the ammonium acetate salt and acetate counterions was related to decreases in the pH of the reconstituted solutions during storage. As shown in Table 5, the pH of the reconstituted solution of Formulation 7 was below 7.5 after one month of storage at 25 °C/60% RH. This corresponds to the molar ratio of acetate to CSP7 in Formulation 7, which decreased after one month of storage at 25 °C/60% RH. In contrast, the molar ratio of acetate to CSP7 in Formulations 14 and 22 remained unchanged after storage, which agrees with the pH of the reconstituted solutions that did not change. When the pH of reconstituted solutions was less than 7.5, the CSP7 concentration (0.5 mg·ml^−1^) is higher than its solubility, thereby inducing peptide aggregation.

This study points out that physical stability of peptide can be affected by the loss of counterion which should be investigated during biological drug development. Peptide aggregation is the process by which two or more peptide molecules associate into larger species consisting of multiple polypeptide chains, which results from the noncovalent association of polypeptide chains or the formation of covalent linkage between chains [43]. Although in our case the soluble aggregates could be redissolved using pH adjustment with a base, soluble aggregates can cause undesirable effects. Soluble aggregate formation can continue until the aggregates are too large and cannot remain soluble in solution [17]. Eventually, insoluble aggregates can be formed. The presence of aggregates can induce immune response in patients, which can affect the safety and efficacy of biopharmaceuticals [44].

### 4.3. Formulation and Process Design to Preserve Counterion Level after Lyophilization

Since the loss of counterions affects the stability of the lyophilized CSP7 acetate, our study evaluated a new strategy to preserve counterion in lyophilized products. The pH modifier was the most influential excipient that affected the loss of counterions. Ammonium hydroxide is a weak base that is more preferably used in peptide formulations, since it does not change the ionic strength of the peptide solution, as compared to sodium hydroxide. Although ammonium hydroxide is a volatile base, and its interaction with acetate counterions causes a pH shift during storage, the optimization of the bulking agent and the lyophilization process help preserve the volatile compounds.

Different types of bulking agent and different molar ratios of CSP7 to the bulking agent affect the presence of acetate counterions in the lyophilized samples. We found that lactose and trehalose can preserve acetate counterions during lyophilization. A higher content of lactose and trehalose resulted in higher molar ratios of acetate to CSP7 in lyophilized CSP7 acetate. Moreover, there was no change in the molar ratio of acetate to CSP7 in lyophilized CSP7-lactose (Formulation 14) and CSP7-trehalose (Formulation 22) after storage at 25 °C/60% RH, and 5 °C, while lyophilized CSP7-mannitol (Formulations 7 and 34) showed a decrease in the molar ratio of acetate to CSP7 after storage at 25 °C/60% RH (Figure 13).

Additionally, the ability of lactose and trehalose to preserve volatile acetate ions was confirmed and shown in Figure 14. We found that lactose and trehalose can preserve more acetate ions than mannitol. However, the ability of lactose and trehalose to preserve counterions has limitations, as the molar ratio of acetate to CSP7 reached a plateau when 21% *v/v* acetic acid was added (Figure 11). In contrast, mannitol-based formulations showed a slight increase in the molar ratio of acetate to CSP7 when a high concentration of acetic acid (14% and 21% *w/w* of bulk CSP7) was added to lyophilized CSP7-mannitol (Figure 11).

We hypothesized that the physical state of the bulking agent is associated with the ability to preserve counterions. As shown in Figure 9, lactose and trehalose were amorphous, while mannitol was crystalline after lyophilization. It is possible that ammonium acetate phase separated from mannitol and was excluded from crystalline freeze concentrate and crystallized completely from the frozen mixture, while ammonium acetate is found in an amorphous glassy matrix of lactose and trehalose. As a result, the loss of acetate ions can be minimized in the amorphous glassy matrix. Although lyophilized 1:5-CSP7 acetate:lactose and 1:5-CSP7:trehalose were amorphous, there were decreases in the molar ratios of acetate to CSP7 after lyophilization. This is possibly due to low solid content in the lyophilized formulations. Low solid content of additives can modify ice crystallization, which is a more open ice structure. Subsequently, the sublimation rate increases [45,46]. Moreover, the solid content in the product also affects the mass transfer of gas during sublimation. High solid content results in high resistance of the dried cake to the vapor flow [47].

The sublimation rate of ammonium acetate was investigated during drying at various chamber pressures. Higher vacuum pressures show that a higher molar ratio of acetate to CSP7 remained in the lyophilized samples. The partial difference between the vapor pressure of water at the subliming ice surface and the chamber pressure provides the driving force for sublimation [48]. Based on Equation (7) [48], the high chamber pressure steps result in the lower rate of sublimation [48]. Therefore, the higher molar ratio of acetate to CSP7 in lyophilized CSP7 prepared at 350 mTorr result from the lower sublimation rate.

(7) $$\frac{\mathrm{dm}}{\mathrm{dt}}=\;\frac{\left(P_{o}-P_{c}\right)}{\left(R_{s}+R_{p}\right)}$$

where $\frac{dm}{dt\;}$ = rate of sublimation, g/cm^2^/hr, *P_O_* = vapor pressure of ice at the product temperature, mmHg, *Pc* = chamber pressure, mmHg, Rp = product resistance (cm^2^ mm Hg hr g^−1^), Rs = stopper resistance (cm^2^ mm Hg hr g^−1^)

### 4.4. Stabilizing Effect of Bulking Agents and Buffers against Peptide Aggregation

We found that different bulking agents exhibited different stabilizing effects on peptide aggregation. As shown in Figure 6, peptide aggregates were found in DPBS after 6 h of shaking on an orbital shaker at 5 °C and 25 °C. The level of aggregates in DPBS was higher after shaking at 25 °C than at 5 °C. These results indicate that the solubility of the peptide was affected by temperature. Chi et al. reported that incubating proteins and peptide solutions at high temperatures can increase the thermal kinetic energy of reactants. This increases the probability of collisions that have enough energy to overcome the activation energies, which subsequently increases the probability of protein and peptide aggregation. These results also indicate that different buffers have different stabilizing effects against mechanical agitation. It was reported that the buffer type and the concentration have an impact on the stability of peptides [49].

Taha et al. showed that tris(hydroxymethyl)aminomethane (TRIS) has favorable interactions with the peptide backbone (–CH_2_COO–NH–) [50]. TRIS is an amine-based buffer in which its –OH and amine groups can interact with BSA mainly through hydrogen bonding [50]. Consequently, the interaction of TRIS with BSA can stabilize the protein molecules [50]. Accordingly, we hypothesized that hydroxyl groups of TRIS may interact with peptide bonds between amino acid residues. Therefore, the interactions between the peptide molecules can be minimized, thus decreasing the probability of aggregate formation.

In the lyophilized state, the stabilizing effect of lactose, trehalose, and mannitol were compared after exposure to 25 °C/75% RH. No CSP7 multimers or degradation products were found in any CSP7 aggregates. This agrees with the study of Hengsawas et al. that reported that the aggregation of CSP7 TFA was not affected the chemical stability [22]. The formation of peptide aggregates was induced by moisture in all lyophilized CSP7 acetate formulations. CSP7 acetate consists of four threonine molecules, which is reported as a strong water-binding residue, so the peptide molecules are likely to absorb moisture when they are exposed to high humidity [51].

Moisture has a detrimental effect on the stability of proteins and peptides because it dramatically increases their mobility [51]. As a result, the peptide is likely to agglomerate, followed by peptide aggregation [51]. Levels of aggregates in lyophilized CSP7-lactose and CSP7-trehalose formulations after exposure to 75% RH were lower than lyophilized CSP7-mannitol (Figure 8). Additionally, Formulation 7 was physically unstable after one month of storage at 25 °C/60% RH, while Formulations 14 and 22 were stable after storage at 25 °C/60% RH up to three months. These results indicate that lactose and trehalose have greater stabilizing effects than mannitol.

Several studies have reported that sugars and polyols can stabilize proteins and peptides during drying and storage. This occurs through two main mechanisms, including the vitrification theory and the water replacement theory [52,53]. According to the water replacement theory, the hydrogen bonding between proteins and water is critical for the thermostability of proteins. Since water is removed during the drying process, the loss of hydrogen bonding between proteins and water can disturb the equilibrium between the native and unfolded forms of the proteins, thereby leading to chemical degradation and aggregation [52]. Sugars and polyols can reportedly form hydrogen bonds with the surface of protein molecules and substitute the hydrogen bonding between proteins and water [52]. The vitrification theory states that the dilution of proteins and peptides in a rigid, amorphous, glassy sugar matrix helps restrict the mobility of proteins and slow down the degradation of proteins during storage [52,53].

Based on both mechanisms, the physical state of sugars and polyols plays an important role in the interaction and miscibility between proteins/peptides and stabilizers [54]. Izutsu et al. reported that the molecular interaction between amorphous sugars or polyols is required to stabilize proteins and peptides during lyophilization [55]. Our observation is consistent with the literature reporting that mannitol shows less capability for stabilizing proteins and peptides due to its crystallization during freeze drying and storage [52]. Crystallization is reported to remove the stabilizer from the protein or peptide phase, which causes less molecular interaction with proteins and peptides [55,56]. Moreover, these results show that the molar ratio of CSP7 to lactose or trehalose has a significant effect on peptide stability. As the content of lactose and trehalose increases, the level of aggregates decreases. Similar observations have been reported by Cleland et al., who found that a minimal specific molar ratio of sugar to protein is required to provide sufficient interaction between proteins/peptides and to provide stabilization against peptide aggregation during storage [57]. In addition to the interaction between sugars and proteins/peptides, amorphous lactose and trehalose are reported as internal desiccants that can absorb water in the drug microenvironment and thereby minimize the free water that can interact with proteins/peptides [58]. As a result, significantly fewer peptide/protein aggregates are formed during storage [58].

## 5. Conclusions

We found that volatile compounds can be sublimated during lyophilization. Excipients (e.g., pH modifiers, bulking agents, buffers) affected the presence of counterions in our lyophilized samples. The pH modifier was the most influential excipient on the loss of counterions, since the modifier can interact with free acetate counterions and subsequently form a volatile salt. The loss of volatile compounds during lyophilization and during storage led to a pH shift in the reconstituted solutions, which affected the physical stability of the lyophilized formulations. The higher content of lactose or trehalose, as well as optimized vacuum pressure during lyophilization, preserved counterions in the lyophilized products and thus maintain their stability.

## Figures and Tables

**Table 1:** Compositions of lyophilized formulations.

Formation No.	Concentration of CSP7	Counterion of CSP7	Bulking Agent	Molar Ratio of CSP7 to Bulking	Buffer	pH Modifier	Vacuum Pressure (mTorr)
**1**	0.5 mg/mL	TFA	-	-	DPBS	NH_4_OH	100
**2**	0.5 mg/mL	TFA	Mannitol	1:140	DPBS	NH_4_OH	100
**3**	0.5 mg/mL	Acetate	-	-	DPBS	NH_4_OH	100
**4**	0.5 mg/mL	Acetate	-	-	TBS	NH_4_OH	100
**5**	0.5 mg/mL	Acetate	Mannitol	1:5	DPBS	NH_4_OH	100
**6**	0.5 mg/mL	Acetate	Mannitol	1:70	DPBS	NH_4_OH	100
**7**	0.5 mg/mL	Acetate	Mannitol	1:140	DPBS	NH_4_OH	100
**8**	0.5 mg/mL	Acetate	Mannitol	1:320	DPBS	NH_4_OH	100
**9**	0.5 mg/mL	Acetate	Mannitol	1:5	TBS	NH_4_OH	100
**10**	0.5 mg/mL	Acetate	Mannitol	1:70	TBS	NH_4_OH	100
**11**	0.5 mg/mL	Acetate	Mannitol	1:140	TBS	NH_4_OH	100
**12**	0.5 mg/mL	Acetate	Mannitol	1:320	TBS	NH_4_OH	100
**13**	0.5 mg/mL	Acetate	Lactose	1:5	DPBS	NH_4_OH	100
**14**	0.5 mg/mL	Acetate	Lactose	1:70	DPBS	NH_4_OH	100
**15**	0.5 mg/mL	Acetate	Lactose	1:140	DPBS	NH_4_OH	100
**16**	0.5 mg/mL	Acetate	Lactose	1:320	DPBS	NH_4_OH	100
**17**	0.5 mg/mL	Acetate	Lactose	1:5	TBS	NH_4_OH	100
**18**	0.5 mg/mL	Acetate	Lactose	1:70	TBS	NH_4_OH	100
**19**	0.5 mg/mL	Acetate	Lactose	1:140	TBS	NH_4_OH	100
**20**	0.5 mg/mL	Acetate	Lactose	1:320	TBS	NH_4_OH	100
**21**	0.5 mg/mL	Acetate	Trehalose	1:5	DPBS	NH_4_OH	100
**22**	0.5 mg/mL	Acetate	Trehalose	1:70	DPBS	NH_4_OH	100
**23**	0.5 mg/mL	Acetate	Trehalose	1:140	DPBS	NH_4_OH	100
**24**	0.5 mg/mL	Acetate	Trehalose	1:320	DPBS	NH_4_OH	100
**25**	0.5 mg/mL	Acetate	Trehalose	1:5	TBS	NH_4_OH	100
**26**	0.5 mg/mL	Acetate	Trehalose	1:70	TBS	NH_4_OH	100
**27**	0.5 mg/mL	Acetate	Trehalose	1:140	TBS	NH_4_OH	100
**28**	0.5 mg/mL	Acetate	Trehalose	1:320	TBS	NH_4_OH	100
**29**	0.5 mg/mL	Acetate	-	-	DPBS	NH_4_OH	350
**30**	0.5 mg/mL	Acetate	Mannitol	1:140	DPBS	NH_4_OH	350
**31**	0.5 mg/mL	Acetate	Lactose	1:70	DPBS	NH_4_OH	350
**32**	0.5 mg/mL	Acetate	Trehalose	1:70	DPBS	NH_4_OH	350
**33**	0.5 mg/mL	Acetate	-	-	DPBS	NaOH	100
**34**	0.5 mg/mL	Acetate	Mannitol	1:140	DPBS	NaOH	100
**35**	0.5 mg/mL	Acetate	Lactose	1:70	DPBS	NaOH	100
**36**	0.5 mg/mL	Acetate	Trehalose	1:70	DPBS	NaOH	100

**Table 2:** Lyophilization parameters used in our study (Adapted from [22]).

Step	Mode	Rate (°C /min)	Temperature (°C)	Vacuum Pressure (mTorr)	Time (min)
Load	Hold	-	5	-	60
Freeze	Ramp	0.5	−55	-	120
	Hold	-	−55	-	120
Anneal	Ramp	0.5	−15	-	80
	Hold	-	−15	-	120
Freeze	Ramp	0.5	−55	-	80
	Hold	-	−55	-	240
Evacuate	Hold	-	−55	100 or 350	30
Primary drying	Ramp	0.1	−30	100 or 350	250
	Hold	-	−30	100 or 350	660
Secondary drying	Ramp	0.08	30	100 or 350	720
	Hold	-	30	100 or 350	240

**Table 3:** Recovery of CSP7 acetate in bulk solution after storage at different temperatures.

Buffer	Time Points (Hours)	Temperature			
		−80 °C	−20 °C	5 °C	25 °C
DPBS	24	100.0 ± 0.1	100.2 ± 0.8	99.9 ± 0.1	100.1 ± 0.1
	48	100.0 ± 0.1	100.3 ± 0.7	99.9 ± 0.1	99.9 ± 0.3
TBS	24	100.1 ± 0.2	100.2 ± 0.2	100.1 ± 0.2	100.2 ± 0.3
	48	100.5 ± 0.4	100.5 ± 0.2	100.1 ± 0.1	100.3 ± 0.1

**Table 4:** Recovery of CSP7 acetate in bulk solution after multiple freeze-thaw cycles.

Buffer	Temperature (°C)	Cycle				
		1	2	3	4	5
DPBS	−20	100.3 ± 0.2	100.7 ± 0.1	100.7 ± 0.1	101.0 ± 0.2	101.3 ± 0.2
	−80	99.8 ± 0.2	100.1 ± 0.1	100.2 ± 0.2	100.4 ± 0.1	100.9 ± 0.1
TBS	−20	99.4 ± 0.3	99.6 ± 0.1	99.7 ± 0.2	99.8 ± 0.2	100.2 ± 0.1
	−80	99.9 ± 0.3	100.3 ± 0.2	100.4 ± 0.2	100.7 ± 0.2	100.6 ± 0.4

**Table 5:** pH of solution after reconstitution of lyophilized formulations.

Formulation No.	Conditions	0 M	1 M	3 M	6 M
2	5 °C	7.72 ± 0.04	-	-	7.63 ± 0.05
	25 °C, 60% RH		7.64 ± 0.05	-	-
7	5 °C	7.60 ± 0.02	-	7.60 ± 0.02	7.56 ± 0.03
	25 °C, 60% RH		7.48 ± 0.03	7.45 ± 0.03	-
14	5 °C	7.84 ± 0.05	-	7.81 ± 0.04	7.83 ± 0.05
	25 °C, 60% RH		7.81 ± 0.03	7.74 ± 0.08	-
22	5 °C	7.81 ± 0.07	-	7.80 ± 0.09	7.81 ± 0.08
	25 °C, 60% RH		7.81 ± 0.04	7.77 ± 0.03	-
34	5 °C	8.06 ± 0.04	-	7.98 ± 0.05	7.94 ± 0.05
	25 °C, 60% RH		8.00 ± 0.07	8.02 ±0.08	-
